# Beyond patient education: Caregiver and family education on improving post-stroke quality of life

**DOI:** 10.51866/lte.1183

**Published:** 2026-07-22

**Authors:** Ebru Kübra Taşpolat, Evrim Coşkun

**Affiliations:** 1 Department of Physical Medicine and Rehabilitation, Elbistan State Hospital, Kahramanmaraş, Turkey.; 2 Department of Physical Medicine and Rehabilitation, Başakşehir Çam and Sakura City Hospital, İstanbul, Turkey.

**Keywords:** Stroke, Caregivers, Patient education, Quality of life, Rehabilitation

**Dear Editor,**

We read with great interest the scoping review titled ‘Patient education for improving the quality of life among community-dwelling survivors of stroke’, recently published in *Malaysian Family Physician.^1^* Stroke remains a major cause of long-term disability worldwide, with substantial consequences for independence, family functioning and community participation.^2^ As clinicians in physical medicine and rehabilitation, we wish to complement the authors’ findings by emphasising the often under-recognised role of caregiver and family education in sustaining post-stroke recovery after discharge.

In rehabilitation practice, functional gains achieved during inpatient care appear more likely to be maintained in the community when primary caregivers receive structured, hands-on education alongside patients. This education should include safe mobilisation, transfer techniques, prevention of falls and pressure injuries, recognition of early warning signs, medication adherence and strategies that promote independence rather than overprotection. Without such preparation, caregivers may unintentionally restrict activity, reinforce dependency or experience progressive burden, all of which may compromise patients’ quality of life and increase the risk of avoidable institutional care.

This clinical observation is supported by emerging evidence. A prospective longitudinal study of matched patient with stroke—caregiver dyads found that caregivers’ preparedness and sense of competence independently predicted favourable changes in patients’ health-related quality of life at discharge.^3^ Similarly, a scoping review of home-based educational interventions for family caregivers of older survivors of stroke reported improvements in caregiver knowledge and skills related to rehabilitation, self-management and communication.^4^ More recently, a systematic review and meta-analysis showed that technology-based psychosocial interventions for caregivers of patients with stroke improved self-efficacy and caregiving competence, particularly when programmes were comprehensive and delivered over several weeks.^5^

Despite this evidence, caregiver education is still inconsistently delivered, rarely standardised and often concentrated around discharge. Future research and clinical guidelines should therefore reposition caregiver and family education as a core, longitudinal component of post-stroke care, not merely as a supplementary discharge activity. Standardised modules could begin during inpatient rehabilitation and be reinforced through primary care and community follow-up. Routine assessment of caregiver burden, self-efficacy and health literacy may also help identify families who require additional support. Family physicians are well positioned to fulfil these roles in the community: They can reinforce discharge education, screen for caregiver burden at follow- up visits and coordinate referrals to community rehabilitation services, thereby bridging the gap between hospital-based rehabilitation and long-term recovery at home.^6^

We congratulate the authors on their timely contribution. Strengthening caregiver education may be a practical and cost-effective way to extend the benefits of patient education, support family-centred recovery and improve quality of life among community-dwelling survivors of stroke.
